# The visual white matter: The application of diffusion MRI and fiber tractography to vision science

**DOI:** 10.1167/17.2.4

**Published:** 2017-02-14

**Authors:** Ariel Rokem, Hiromasa Takemura, Andrew S. Bock, K. Suzanne Scherf, Marlene Behrmann, Brian A. Wandell, Ione Fine, Holly Bridge, Franco Pestilli

**Affiliations:** arokem@uw.edu http://arokem.org; htakemur@nict.go.jp; bock.andrew@gmail.com; suzyscherf@psu.edu; behrmann@cmu.edu; wandell@stanford.edu; ionefine@uw.edu; holly.bridge@ndcn.ox.ac.uk; franpest@indiana.edu http://francopestilli.com; The University of Washington eScience Institute, Seattle, WA, USA; Center for Information and Neural Networks (CiNet), National Institute of Information and Communications Technology, and Osaka University, Suita-shi, Japan; Graduate School of Frontier Biosciences, Osaka University, Suita-shi, Japan; University of Pennsylvania, Philadelphia, PA, USA; Penn State University, State College, PA, USA; Carnegie Mellon University, Pittsburgh, PA, USA; Stanford University, Stanford, CA, USA; University of Washington, Seattle, WA, USA; Oxford University, Oxford, UK; Indiana University, Bloomington, IN, USA

**Keywords:** *MRI*, *diffusion MRI*, *brain*, *white matter*, *brain connectivity*, *visual cortex*, *visual disability*, *visual development*, *categorical perception*, *face perception*, *software*, *computational modeling*

## Abstract

Visual neuroscience has traditionally focused much of its attention on understanding the response properties of single neurons or neuronal ensembles. The visual white matter and the long-range neuronal connections it supports are fundamental in establishing such neuronal response properties and visual function. This review article provides an introduction to measurements and methods to study the human visual white matter using diffusion MRI. These methods allow us to measure the microstructural and macrostructural properties of the white matter in living human individuals; they allow us to trace long-range connections between neurons in different parts of the visual system and to measure the biophysical properties of these connections. We also review a range of findings from recent studies on connections between different visual field maps, the effects of visual impairment on the white matter, and the properties underlying networks that process visual information supporting visual face recognition. Finally, we discuss a few promising directions for future studies. These include new methods for analysis of MRI data, open datasets that are becoming available to study brain connectivity and white matter properties, and open source software for the analysis of these data.

## Introduction

The cerebral hemispheres of the human brain can be subdivided into two primary tissue types: the white matter and the gray matter (Fields, 2008a). Whereas the gray matter contains neuronal cell bodies, the white matter contains primarily the axons of these neurons and glial cells. The axons constitute the connections that transmit information between distal parts of the brain: millimeters to centimeters in length. Some types of glia (primarily oligodendrocytes) form an insulating layer around these axons (myelin sheaths) that allow the axons to transmit information more rapidly (Waxman & Bennett, 1972) and more accurately (J. H. Kim, Renden, & von Gersdorff, 2013) and reduces the energy consumption used for long-range signaling (Hartline & Colman, 2007).

Much of neuroscience has historically focused on understanding the functional response properties of individual neurons and cortical regions (Fields, 2004, 2013). It has often been implicitly assumed that white matter and the long-range neuronal connections it supports have a binary nature: either they are connected and functioning or they are disconnected. More recently, there is an increasing focus on the roles that the variety of properties of the white matter may play in neural computation (Bullock et al., 2005; Jbabdi et al., 2015; Reid, 2012; Sporns, Tononi, & Kötter, 2005) together with a growing understanding of the importance of the brain networks composed of these connections in cognitive function (Petersen & Sporns, 2015).

The renewed interest in white matter is due in part to the introduction of new technologies. Long-range connectivity between parts of the brain can be measured in a variety of ways. For example, fMRI is used to measure correlations between the blood oxygenation level–dependent (BOLD) signal in different parts of the brain. In this review, we focus on results from diffusion-weighted MRI (dMRI). Together with computational tractography, dMRI provides the first opportunity to measure white matter and the properties of long-range connections in the living human brain. We sometimes refer to the estimated connections as “fascicles,” an anatomical term that refers to bundles of nerve or muscle fibers. Measurements in living brains demonstrate the importance of white matter for human behavior, health, and disease (Fields, 2008b; Jbabdi et al., 2015; Johansen-Berg & Behrens, 2009); for development (Lebel et al., 2012; Yeatman, Wandell, & Mezer, 2014); and for learning (Bengtsson et al., 2005; Blumenfeld-Katzir, Pasternak, Dagan, & Assaf, 2011; Hofstetter, Tavor, Moryosef, & Assaf, 2013; Sagi et al., 2012; Sampaio-Baptista et al., 2013; Thomas & Baker, 2013). The white matter comprises a set of active fascicles that respond to human behavior by adapting their density, their shape, and their molecular composition in a manner that corresponds to human cognitive, motor, and perceptual abilities.

**Figure 1:**
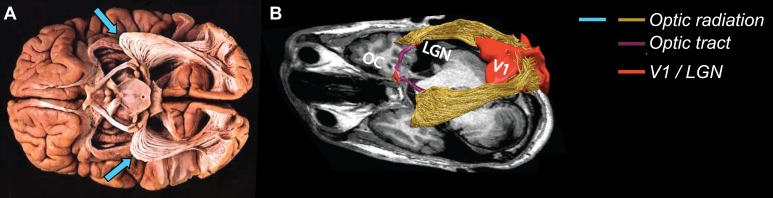
Postmortem and in vivo study of the visual white matter. (A) Postmortem dissection of the optic radiation (OR), showing the Meyer's loop bilaterally (blue arrows; adapted from Sherbondy et al., 2008b). (B) In vivo dissection of the optic radiation showing the optic chiasm, Meyer's loop, and the optic tract as well as both primary visual cortex (V1) and the LGN (Ogawa et al., 2014).

The visual white matter sustains visual function. In the primary visual pathways, action potentials travel from the retina via the optic nerve, partially cross at the optic chiasm, and continue via the optic tract to the lateral geniculate nucleus (LGN) of the thalamus. From the LGN, axons carry visual information laterally through the temporal lobe, forming the structure known as Meyer's loop, and continue to the primary visual cortex (Figure 1). The length of axons from the retina to the LGN is approximately 5 cm, and the length of the optic radiation from the anterior tip of Meyer's loop to the calcarine sulcus is approximately 10 cm with an approximate width of 2 cm at the lateral horn of the ventricles (Peltier, Travers, Destrieux, & Velut, 2006; Sherbondy, Dougherty, Napel, & Wandell, 2008). Despite traveling the entire length of the brain, responses to visual stimuli in the cortex arise very rapidly (Maunsell & Gibson, 1992), owing to the high degree of myelination of the axons in the primary visual pathways. Healthy white matter is of crucial importance in these pathways as fast and reliable communication of visual input is fundamental for healthy visual function.

This review presents the state of the art in dMRI studies of the human visual system. We start with an introduction of the measurements and the analysis methods. Following that, we review three major applications of dMRI:

1. The delineation of the relationship between visual field maps and the white matter connections between the maps. This section focuses on a test case of the connections between dorsal and ventral visual field maps.
2. The effects of disorders of the visual system on the visual white matter. This section focuses on the changes that occur in the white matter in cases of peripheral and cortical blindness and on white matter connectivity that underlies cross-modal plasticity and residual vision, respectively, in these cases.
3. The role that the visual white matter plays in visual discrimination of specific categories of visual objects. In particular, this section focuses on the white matter substrates of face perception.

Taken together, these three sections present multiple facets of dMRI research in vision science, covering a wide array of approaches and applications. They all demonstrate the ways in which studying the white matter leads to a more complete understanding of the biology of the visual system: its structure, response properties, and relation to perception and behavior. In the summary and conclusions of this review, we point out some common threads among these applications, point to a few of the challenges facing the field, and the promise of future developments to help address some of these challenges.

## Methods for in vivo study of the human white matter

### Measuring white matter using dMRI

dMRI measures the directional diffusion of water in different locations in the brain. In a dMRI experiment, a pair of magnetic field gradient pulses is applied. The first pulse tips the orientation of the spins of water molecules and causes dispersion of their phases. The second pulse—of the same magnitude but opposite direction—refocuses the signal by realigning the phases. Refocusing will not be perfect for protons that have moved during the time between the gradient pulses, and the resulting loss of signal is used to infer the mean diffusion distance of water molecules in the direction of the magnetic gradient. Measurements conducted with gradients applied along different directions sensitize the signal to diffusion in these directions (Figure 1B).

The sensitivity of the measurement to diffusion increases with the amplitude and duration of the magnetic field gradients as well as with the time between the two gradient pulses (the *diffusion time*). These three parameters are usually summarized in a single number: the b-value. Increased sensitivity to diffusion at higher b-values comes at a cost: Measurements at higher b-values also have lower signal-to-noise ratio. In the typical dMRI experiment, diffusion times are on the order of 50–100 ms. Within this timescale, freely moving water molecules may diffuse an average of approximately 20 μm (at body temperature; Le Bihan & Iima, 2015). This diffusion distance is found in the ventricles of the brain, for example. Within other brain regions, the average diffusion distance is reduced by brain tissue barriers (Figure 1).

**Figure 2:**
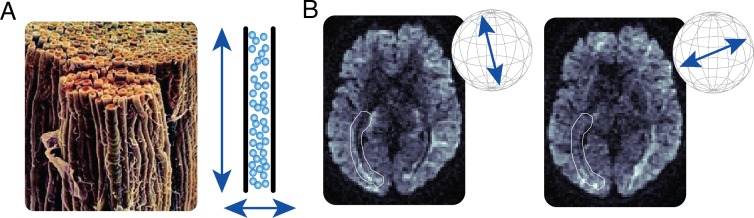
Inferences about white matter from measurements of water diffusion. (A) Micrograph of the human optic nerve. Left, bundles of myelinated axonal fascicles from the human optic nerve (image courtesy of Dr. George Bartzokis). Right, cartoon example of anisotropic water diffusion restriction by white matter fascicles. Water diffuses further along the direction of the “tube” (Pierpaoli & Basser, 1996). (B) Example of diffusion-weighted magnetic resonance measurements in a single slice of a living human brain. The same brain slice is shown as imaged with two different diffusion weighting directions. Diffusion weighting directions are shown by the inset arrows. The white highlighted area indicates the approximate location of part of the optic radiation (OR). The longitudinal shape of the OR appears as a local darkening of the MRI image when the diffusion-weighting gradient is aligned with the direction of the myelinated fascicles within the OR (right-hand panel).

In regions of the brain or body that contain primarily spherically shaped cell bodies, water diffusion is equally restricted in all directions: it is isotropic. On the other hand, in fibrous biological structures, such as muscle or nerve fascicles, the diffusion of water is restricted across the fascicles (e.g., across axonal membranes) more than along the length of the fascicles (e.g., within the axoplasm; Figure 2A). In these locations, diffusion is anisotropic. Measurements of isotropic and anisotropic diffusion can therefore be used to estimate the microstructural properties of brain tissue and brain connectivity.

Inferring brain tissue properties and connectivity from dMRI data usually involves two stages: The first estimates the microstructure of the tissue locally in every voxel. The second stage connects local estimates across voxels to create a macrostructural model of the axonal pathways. We briefly review these methods in the sections that follow (see also Wandell, 2016).

### White matter microstructure

**Figure 3:**
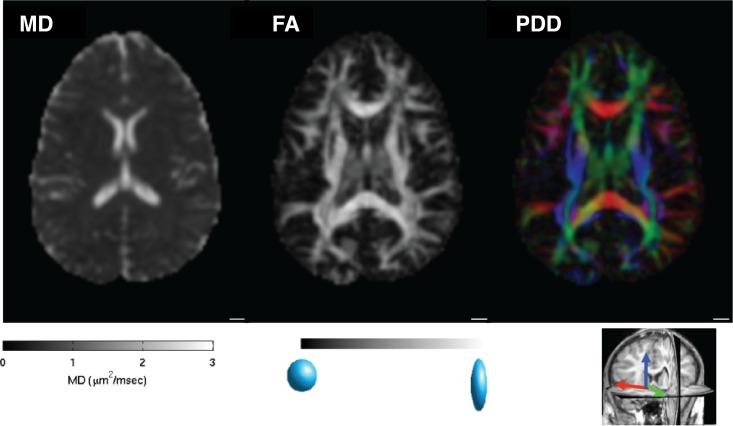
Diffusion tensor imaging. The figure shows the three primary estimates of white matter organization derived from the diffusion tensor model (DTM; Basser, Mattiello, & LeBihan, 1994a, 1994b) measured in a single slice of a human brain. Mean diffusivity (MD; left) represents water diffusion averaged across all measured diffusion directions. Fractional anisotropy (FA; middle) represents the variability of diffusion in different directions. This value is unitless and bounded between zero and one, and it is highest in voxels containing a single dense fascicle, such as in the corpus callosum. The principal diffusion direction (PDD; right) is the direction of maximal diffusion in each voxel. It is often coded with a mapping of the *X*, *Y*, and *Z* components of the direction vector mapped to red, green, and blue color channels, respectively, and scaled by FA. Main white matter structures, such as the corpus callosum (red, along the right-to-left *x*-axis) and the corticospinal tract (blue, along the superior–inferior *y*-axis) are easily detected in these maps.

Even though dMRI samples the brain using voxels at millimeter resolution, the measurement is sensitive to diffusion of water within these voxels at the scale of micrometers. This makes dMRI a potent probe of the aggregate microstructure of the brain tissue in each voxel. The diffusion tensor model (DTM; Basser, Mattiello, & LeBihan, 1994a, 1994b) approximates the directional profile of water diffusion in each voxel as a 3-D Gaussian distribution. The direction in which this distribution has its largest variance is an estimate of the principal diffusion direction (PDD; Figure 3): the direction of maximal diffusion. In certain places in the brain, the PDD is aligned with the orientation of the main population of nerve fascicles and can be used as a cue for tractography (see below). In addition to the PDD, the DTM provides other statistics of diffusion: the mean diffusivity (MD), which is an estimate of the average diffusivity in all directions, and the fractional anisotropy (FA), which is an estimate of the variance in diffusion in different directions (Pierpaoli & Basser, 1996; Figure 3). These statistics are useful for characterizing the properties of white matter, and numerous studies have shown that variance in these statistics within relevant white matter tracts can predict individual differences in perception (Genç et al., 2011), behavioral and cognitive abilities (e.g., Yeatman et al., 2011), and mental health (Thomason & Thompson, 2011). A major advantage of the DTM is that it can be estimated from relatively few measurement directions: accurate (Rokem et al., 2015) and reliable (Jones, 2004) estimates of the tensor parameters require measurements in approximately 30–40 directions, which requires approximately 10 min in a standard clinical scanner.

The interpretation of tensor-derived statistics is not straightforward. MD has been shown to be sensitive to the effects of stroke during its acute phases (Mukherjee, 2005), and loss of nerve fascicles due to Wallerian degeneration and demyelination also results in a decrease in FA (Beaulieu, Does, Snyder, & Allen, 1996) because of loss of density and myelin in brain tissue. But FA also decreases in voxels in which there are crossing fascicles (Frank, 2001, 2002; Pierpaoli & Basser, 1996), rendering changes in FA ambiguous. A recent study estimated that approximately 90% of the white matter contains fascicle crossings (Jeurissen, Leemans, Tournier, Jones, & Sijbers, 2013). Therefore, it is unwarranted to infer purely from FA that some locations or some individuals have higher or lower “white matter integrity” (Jones, Knösche, & Turner, 2013).

In an analogous issue, one limitation of the DTM that was recognized early on (Pierpaoli & Basser, 1996) is that it can only represent a single PDD. In voxels with crossing fascicles, the PDD will report the weighted average of the individual fascicle directions rather than the direction of any one of the fascicles (Rokem et al., 2015).

Nevertheless, despite its limitations, the statistics derived from the DTM provide useful information about tissue microstructure. This model has been so influential that diffusion tensor imaging or DTI is often used as a synonym for dMRI.

**Figure 4:**
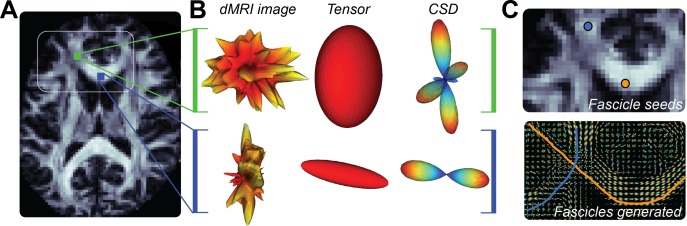
Relationship between dMRI signals, voxel models of diffusion, and tractography. (A) An axial brain slice. Two voxel locations are indicated in blue and green. The white rectangle indicates the location of the images in panel C. (B) Measured diffusion-weighted MRI in the colored locations in panel A. Left column: Diffusion signal for the green (top) and blue (bottom) locations rendered as 3-D surfaces with the color map indicating the intensity of the diffusion-weighted signal in each direction (red is low signal or high diffusion and yellow-white is high signal or low diffusion). Middle column: The 3-D Gaussian distribution of diffusion distance, estimated from the DTM for the signal to the left. The major axis of the ellipsoid indicates the PDD estimated by the tensor model, different for the two voxels. Right column: fODF estimated by a fascicle mixture model: The constrained spherical deconvolution (CSD) model (Tournier et al., 2007) from the signal to its left. CSD indicates several probable directions of fascicles. Color map indicates likelihood of the presence of fascicles in a direction. (C) Top panel: Detail of the region highlighted in panel A (white frame) with example of two seeds randomly placed within the white matter and used to generate the fascicles in the bottom panel. Bottom panel: Fits of the CSD model to each voxel and two example tracks (streamlines) crossing at the location of the centrum semiovale.

To address these challenges, starting with the work of Larry Frank (Frank, 2001, 2002), there have been a series of models that approximate the dMRI signal in each voxel as a mixture combined from the signals associated with different fascicles within each voxel (Behrens, Berg, Jbabdi, Rushworth, & Woolrich, 2007; Dell'Acqua et al., 2007; Rokem et al., 2015; Tournier, Calamante, & Connelly, 2007; Tournier, Calamante, Gadian, & Connelly, 2004). Common to all these models is that they estimate a fascicle orientation distribution function (fODF or FOD) based on the partial volumes of the different fascicles contributing to the mixture of signals. These models are more accurate than the DTM in regions of the brain where large populations of nerve fascicles are known to intersect (Figure 4) and also around the optic radiations (Alexander, Barker, & Arridge, 2002; Rokem et al., 2015).

A second approach to modeling complex configurations of axons in a voxel are offered by so-called “model-free” analysis methods, such as q-space imaging (Tuch, 2004) and diffusion spectrum imaging (DSI; Wedeen, Hagmann, Tseng, Reese, & Weisskoff, 2005). These analysis methods estimate the distribution of orientations directly from the measurement, using the mathematical relationship between the dMRI measurement and the distribution of diffusion in different directions and without interposing a model of the effect of individual fascicles and the combination of fascicles on the measurement. These approaches typically require a larger number of measurements in many diffusion directions and diffusion weightings (b-values), demanding a long measurement duration.

### White matter macrostructure

**Figure 5:**
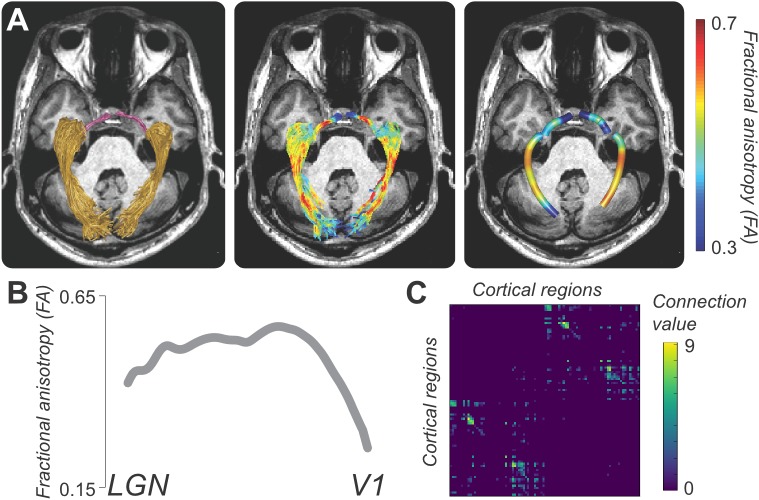
Example applications of tractography to study brain connections and white matter. (A) Estimates of the optic radiation (OR; left panel gold) and tract (OT; left panel, magenta) from probabilistic tractography (Ogawa et al., 2014). Estimates of FA projected on top of the anatomy of the OR and OT (right panel). (B) Measurement of FA averaged across the length of the left optic radiation in panel A. (C) Matrix of connections between several cortical brain areas. The connection value in each cell of the matrix represents the number of fascicles touching the two regions.

Computational tractography refers to a collection of methods designed to make inferences about the macroscopic structure of white matter pathways (Figure 5C; Jbabdi et al., 2015; Wandell, 2016). These algorithms combine models of the local distribution of neuronal fascicle orientations across multiple voxels to track long-range neuronal pathways. The 3-D curves estimated by these algorithms (sometimes referred to as “streamlines”) represent collections of axons (i.e., fascicles), not individual axons: brain “highways” that connect distal brain areas that are millimeters to centimeters apart. Below we describe tractography by dividing it into three major phases of analysis: initiation, propagation, and termination of tracking.

White matter tracking is generally initiated (seeded) in many locations within the white matter volume. In some cases, streamlines are seeded within spatially constrained regions of interest (ROIs). This is the case when the goal is to identify brain connections by tracking between two specific brain areas (Smith, Tournier, Calamante, & Connelly, 2012).

Three major classes of algorithms are used to propagate streamlines: deterministic, probabilistic, and global. *Deterministic tractography* methods take the fascicle directions estimated in each voxel at face value and draw a streamline by following the principal fascicle directions identified by the voxel-wise model fODF (Conturo et al., 1999; Mori, Crain, Chacko, & van Zijl, 1999; Mori & van Zijl, 2002). *Probabilistic tractography* methods (Behrens et al., 2003; Behrens et al., 2007; Tournier, Calamante, & Connelly, 2012) accept the voxel-wise estimate of the fODF but recognize that such estimates may come with errors. Instead of strictly following the directions indicated by the fODF, they consider the fODF to be a probability distribution of possible tracking directions. These methods generate streamlines aligned with the principal fiber directions with higher probability, and they also generate streamlines away from the principal directions with nonzero probability. *Global tractography* methods build streamlines that conform to specified “global” properties or heuristics (Mangin et al., 2013; Reisert et al., 2011). For example, some algorithms constrain streamlines to be spatially smooth (Aganj et al., 2011).

Tractography is generally restricted to the white matter volume, so when streamlines reach the end of the white matter, they are terminated. To determine the extent of the white matter, methods of tissue segmentation based on anatomical measurements (Fischl, 2012) are used or differences in diffusion properties between different tissue types. For example, because gray matter has low FA, tracking is often terminated when FA drops below a threshold.

Tractography reliably identifies certain large, major white matter tracts (Wakana et al., 2007; Yeatman, Dougherty, Myall, Wandell, & Feldman, 2012), but some fundamental properties of the white matter anatomy are still a matter of debate (for example, see Catani, Bodi, & Dell'Acqua, 2012; Wedeen et al., 2012a, 2012b). This is partially because of the diversity of dMRI methods and because of the challenge to validate the results of different tractography solutions against a gold standard. Several approaches have been proposed to tractography validation: Postmortem validation methods show the degree to which tractography identifies connections found by tracing and histological methods. Validation with these methods is complicated by the fact that the histological methods have their own limitations (Bastiani & Roebroeck, 2015; Goga & Türe, 2015; Simmons & Swanson, 2009). Another approach uses phantoms of known structure or simulations based on physical models (Côté et al., 2013). Alternatively, methods that directly estimate the error of a tracking method with respect to the MRI data have also been proposed (Daducci, Palu, & Thiran, 2015; Pestilli et al., 2014; Sherbondy, Dougherty, Ben-Shachar, Napel, & Wandell, 2008; Smith, Tournier, Calamante, & Connelly, 2015). These methods rely on a forward model approach: The estimated tracts are used to generate a synthetic version of the measured diffusion signal and to compute the tractography model error by evaluating whether the synthetic and measured data match (i.e., cross-validation).

### Analysis of white matter tracts and brain connections

Once white matter tracts and brain connections have been identified using tractography, these estimates have traditionally been analyzed in three ways:

1. Identify major white matter pathways (Catani, Howard, Pajevic, & Jones, 2002; Catani & Thiebaut de Schotten, 2008). One typical step after tractography is to cluster these curves together into groups (Garyfallidis, Brett, Correia, Williams, & Nimmo-Smith, 2012; Wassermann, Bloy, Kanterakis, Verma, & Deriche, 2010) and align these curves to each other, either across different individuals, across hemispheres (Garyfallidis, Ocegueda, Wassermann, & Descoteaux, 2015), or to standard anatomical landmarks (Wakana et al., 2007; Yeatman et al., 2012; Yendiki et al., 2011). For example, the optic radiation (Kammen, Law, Tjan, Toga, & Shi, 2015; Sherbondy, Dougherty, Napel, et al., 2008) and other connections between the thalamus and visual cortex (Ajina, Pestilli, Rokem, Kennard, & Bridge, 2015; Allen, Spiegel, Thompson, Pestilli, & Rokers, 2015) can systematically be identified in different individuals based on their end points.
2. Estimate microstructural tissue properties (such as FA and MD) within white matter tracts (Yeatman et al., 2012; Yendiki et al., 2011; see Figure 5A, B).
3. Estimate connectivity between different regions of the cortex (Jbabdi et al., 2015; Rubinov, Kötter, Hagmann, & Sporns, 2009; Figure 5C).

Individual estimates of microstructure and connectivity correspond to behavior, but they can also be combined across individuals to compare between groups (e.g., patients and controls). Below, we present several cases in which group analyses have been applied to study visual function.

## Tracts and connections across human visual maps

The spatial arrangement of retinal inputs is maintained in the visual cortex; signals from nearby locations in the retina project to nearby locations in the cortex (Henschen, 1893; Holmes & Lister, 1916; Inouye, 1909; Wandell, Dumoulin, & Brewer, 2007; Wandell & Winawer, 2011). Vision scientists routinely use fMRI to identify these visual field maps (DeYoe, Bandettini, Neitz, Miller, & Winans, 1994; Dumoulin & Wandell, 2008; Engel et al., 1994; Sereno et al., 1995), and more than 20 maps have been identified to date (Amano, Wandell, & Dumoulin, 2009; Arcaro, McMains, Singer, & Kastner, 2009; Brewer, Liu, Wade, & Wandell, 2005; Larsson & Heeger, 2006; Press, Brewer, Dougherty, Wade, & Wandell, 2001; Silver & Kastner, 2009; Smith, Greenlee, Singh, Kraemer, & Hennig, 1998; Wandell & Winawer, 2011). Recent advances in dMRI and tractography are opening new avenues to study connections between visual field maps through the white matter in living brains. This section introduces what we can learn from relating visual field maps to the end points of the white matter pathways.

### Organization of visual maps in humans

The functional organization of the visual field maps in humans and animal models has been identified by convergent knowledge mostly from fMRI, neuropsychological, and cytoarchitectonic studies (Kolster, Janssens, Orban, & Vanduffel, 2014; Wandell et al., 2007; Wandell & Winawer, 2011; Vanduffel, Zhu, & Orban, 2014). Over the last two decades, fMRI studies have made several major contributions toward understanding the organization of visual maps. First, the spatial resolution of fMRI allows measurements of the topographic organization of visual field representations. The ability of fMRI to estimate the border of visual field maps has improved over decades, and now it is possible to identify the borders of V1/V2/V3 even in the foveal visual field (Schira, Tyler, Breakspear, & Spehar, 2009). Second, fMRI provides digital, reproducible datasets of visual responses from the brains of living individuals. Such datasets offer us the opportunity to resolve controversies regarding visual field map definitions that arise from the comparison between anatomical and cytoarchitectonic studies (Kolster et al., 2014). Finally, fMRI measurements also revealed that different visual maps preferentially respond to specific categories of stimulus, such as motion, color, faces, objects, places, and visual word forms (Dubner & Zeki, 1971; Epstein & Kanwisher, 1998; Huk, Dougherty, & Heeger, 2002; Huk & Heeger, 2002; Malach et al., 1995; Movshon, Adelson, Gizzi, & Newsome, 1985; Tootell et al., 1995; Wade, Augath, Logothetis, & Wandell, 2008; Watson et al., 1993), suggesting specific computational roles for different visual areas.

Vision scientists have proposed several theories of the overall organizing principles of the visual cortex. One of the major theories is the “two-stream hypothesis,” which distinguishes dorsal and ventral visual maps (Ungerleider & Haxby, 1994; Ungerleider & Mishkin, 1982; Milner & Goodale, 1995). According to this model, the dorsal stream engages in spatial processing and action guidance whereas the ventral stream engages in analyzing colors and forms. Another organizing principle divides the visual maps into clusters, which share common eccentricity maps (Kolster et al., 2014; Kolster et al., 2009; Wandell et al., 2007).

Although these organizing principles are widely accepted (Kolster et al., 2014; Vanduffel et al., 2014; Wandell & Winawer, 2011), challenges still remain, such as understanding the biological determinants of the large-scale communication across the visual system. We have only limited knowledge of how visual field clusters communicate, and understanding the organization of visual white matter tracts and their relationship to the visual field maps can clarify the functional role of maps and clusters. For example, detailed understanding of the communication between the dorsal and ventral human visual streams is important because visual behavior generally requires integration of spatial and categorical information (e.g., in skilled reading; Vidyasagar & Pammer, 2010). Clarifying the anatomy of the white matter tracts communicating between different divisions of the visual system also opens up new opportunities to study the properties of the visual white matter in relation to development, aging, disease, and behavior (Dagnelie, 2013; Tzekov & Mullan, 2014; Wandell, Rauschecker, & Yeatman, 2012; Yoon, Sheremata, Rokem, & Silver, 2013).

### Combining fMRI and dMRI to study the white matter tracts communicating between visual maps

Studies that combine measurements of dMRI and fMRI in individual participants provide a unique opportunity to compare white matter anatomy with cortical response patterns and understand their relationships. Over the course of the last decade, the quality of dMRI data acquisition and tractography methods has significantly improved (Wandell, 2016). As measurements and methods improve, the studies comparing fMRI-based visual field mapping and white matter tracts have also made progress. Earlier reports used deterministic tractography based on the tensor model to identify white matter tracts in the visual cortex and explored the relationship to visual field maps. Dougherty, Ben-Shachar, Bammer, Brewer, and Wandell (2005) analyzed the relationship between the visual field maps and corpus callosum streamlines. They generated tractography solutions connecting visual cortex and corpus callosum in each hemisphere. Then they classified streamline groups based on which visual maps are near the occipital termination of each fascicle. They estimated that streamlines that originated from dorsal visual maps (such as V3d, V3A/B, and IPS-0) tended to arrive to relatively superior parts of the splenium (the posterior portion of the corpus callosum) whereas streamlines that originated from ventral maps (such as hV4) may arrive at the inferior part of the splenium in a symmetric pattern across the left and right hemispheres. This analysis was subsequently extended to measure the properties of this pathway in relation to reading (Dougherty et al., 2007) and blindness (Bock et al., 2013; Saenz & Fine, 2010; see below). Later, Kim et al. (2006) explored white matter tracts between early visual cortex and category-selective regions by combining fMRI, dMRI, and deterministic tensor-based fiber tractography. They reported the estimates on several white matter tracts, including a tract connecting the primary visual cortex and the parahippocampal place area (PPA; Epstein & Kanwisher, 1998). However, the approaches using the DTM and deterministic tractography have limited ability to model crossing fibers and limited robustness against measurement noise. In fact, Kim et al. (2006) reported that there is a large individual difference in estimated tracts across subjects, which made it hard for them to interpret the estimated structural connections. Dougherty et al. (2005) also mentioned that they missed callosal fibers terminating in ventral visual cortex in some subjects because of the limitation of these methods.

Later studies used more advanced diffusion models to reconstruct fiber pathways in relation to the visual cortex. Greenberg et al. (2012) hypothesized that there should be a white matter tract between the intraparietal sulcus (IPS) and early visual areas (V1/V2/V3), motivated by the studies demonstrating visual field maps in the IPS (Sereno, Pitzalis, & Martinez, 2001; Silver & Kastner, 2009; Silver, Ress, & Heeger, 2005; Swisher, Halko, Merabet, McMains, & Somers, 2007). The IPS maps do not share the common foveal confluence with V1/V2/V3, but these maps do have similar angular representation as V1/V2/V3. Thus, it is reasonable to hypothesize that there should be a white matter tract connecting directly between V1/V2/V3 and IPS maps, according to the theory of foveal clusters (Wandell et al., 2007). To test this hypothesis, they used DSI to resolve crossing fibers within the the occipital and parietal lobes. They then generated tractography solutions seeding in the fMRI-based visual field maps (V1/V2/V3, anterior and posterior IPS) and evaluated the connectivity between maps by counting the number of streamlines connecting maps, scaled by the distance between the maps. This analysis demonstrated that there is a tract connecting IPS and earlier maps (V1/V2/V3), and streamlines in this tract have a tendency to connect areas with similar visual field coverage.

**Figure 6:**
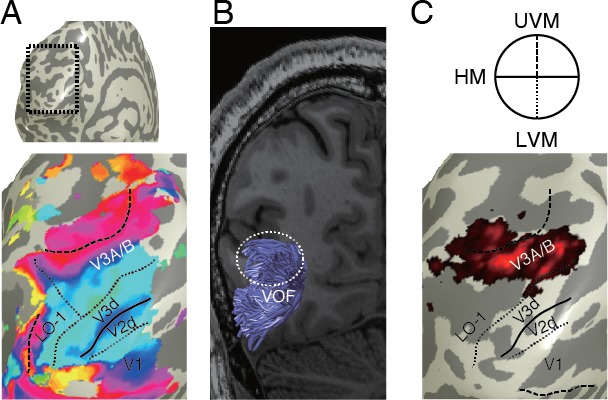
Relationship between cortical visual field maps and tract end points. (A) Visual field maps identified by fMRI. The surface of the dorsal visual cortex was extracted (dotted box in top panel) and enlarged (bottom panel). Colors on the bottom panels describe polar angle representation in population receptive field maps (Dumoulin & Wandell, 2008; red: upper vertical meridian, blue: horizontal, green: lower vertical meridian). The boundaries between visual field maps are defined as polar angle reversals in population receptive field maps. (B) The VOF identified in an identical subject using dMRI and fiber tractography (reproduced from Takemura, Rokem, et al., 2016, with permission). (C) Overlay between visual field maps and VOF projections. Color maps indicate voxels near the VOF end points in this hemisphere, and the color scale indicates the density of fascicle end points. The borders of visual field maps (solid and dotted lines; upper panel depicts the representation of horizontal and vertical meridians) were adopted from fMRI-based mapping (panel A).

Takemura, Rokem, and colleagues (2016) further extended these methods to associate visual field maps and tractography by separating the generation of the fascicles and evaluation of tractography solutions. Streamlines were generated using probabilistic tractography and constrained spherical deconvolution (Tournier et al., 2007, 2012), and a subset of streamlines was selected based on linear fascicle evaluation (Pestilli et al., 2014). This analysis rejects spurious streamlines that do not explain the diffusion signal and evaluates the statistical evidence for the existence of remaining streamlines. Based on this approach, the vertical occipital fasciculus (VOF) was delineated and validated. The VOF, which connects dorsal and ventral visual field maps, was known to 19th century anatomists from postmortem studies (Déjerine & Déjerine-Klumpke, 1895; Sachs, 1892; Wernicke, 1881), but it was widely ignored in the vision literature until recent studies (Duan, Norcia, Yeatman, & Mezer, 2015; Martino & Garcia-Porrero, 2013; Takemura, Rokem et al., 2016; Weiner, Yeatman, & Wandell, 2016; Yeatman, Weiner et al., 2014; Yeatman, Rauschecker, & Wandell, 2013). By combining fMRI and dMRI, it is possible to visualize the VOF end points near the visual field maps (Takemura, Rokem et al., 2016): The dorsal end points of the VOF are near V3A, V3B, and neighboring maps, and the ventral end points of the VOF are near hV4 and VO-1 (Figure 6; Takemura, Rokem et al., 2016). This is important because it sheds light on the nature of communication through the VOF: hV4 and VO-1 are the first full hemifield maps in the ventral stream (Arcaro et al., 2009; Brewer et al., 2005; Wade, Brewer, Rieger, & Wandell, 2002; Winawer, Horiguchi, Sayres, Amano, & Wandell, 2010; Winawer & Witthoft, 2015), and V3A and V3B are the first visual field maps to contain a full hemifield representation in the dorsal stream. The proximity of the VOF to these visual field maps suggests that it contributes to transfer of the upper and lower visual field representation between dorsal and ventral maps to build hemifield representation in midlevel visual areas. The structure of VOF may also have implications for how dorsal and ventral streams communicate to integrate spatial and categorical information: V3A and V3B are known to be selective for motion and binocular disparity (Ashida, Lingnau, Wall, & Smith, 2007; Backus, Fleet, Parker, & Heeger, 2001; Cottereau, McKee, Ales, & Norcia, 2011; Fischer, Bulthoff, Logothetis, & Bartels, 2012; Goncalves et al., 2015; Nishida, Sasaki, Murakami, Watanabe, & Tootell, 2003; Tootell et al., 1997; Tsao et al., 2003), and hV4 and VO-1 are selective for color (Brewer et al., 2005; Brouwer & Heeger, 2009; Goddard, Mannion, McDonald, Solomon, & Clifford, 2011; McKeefry & Zeki, 1997; Wade et al., 2002; Wade et al., 2008; Winawer & Witthoft, 2015).

### Summary

The evolution of our understanding of the white matter connecting different visual field maps depends closely on the development of dMRI techniques. As seen in the survey of studies presented above, as methods of dMRI and tractography continue to develop, our ability to identify the relationship between white matter tracts and visual field maps has improved, but there are still several challenges that need to be addressed before we can envision creating the full wiring diagrams of white matter tracts connecting visual maps. Hereafter, we discuss the current challenges and future directions. We focus on two of the major issues.

The first challenge is a difficulty in following the visual representation along the length of a white matter tract at the resolution of the streamlines generated using tractography. This is because these streamlines are not models of an individual axon; rather, they are models of large collections of axons. In other mammals, there is evidence showing medially and laterally located axons exchanging positions along the optic radiations (Nelson & Le Vay, 1985) and of the topography of the visual field inverting between LGN and V1 (Connolly & Van Essen, 1984). If this is also the case in human brains, then it may not be possible for a single streamline generated by tractography to preserve the identical visual field representation along the optic radiation. Thus, the interpretation of optic radiation tractography still depends on known anatomy of visual field topography from postmortem studies (Sherbondy, Dougherty, Napel et al., 2008). For the foreseeable future, dMRI-based tractography will be more useful in identifying white matter tracts at a macroscopic scale rather than tracking precise visual field representation in detail.

The second challenge is a limitation in associating fascicle end points in tractography solutions with cortical projections into the gray matter. Tractography is usually terminated at the boundary between gray and white matter because standard dMRI measurements fall under the resolution required to accurately delineate the complex tissue organization within gray matter. Therefore, there is uncertainty in relating tract end points to the cortical surface. This uncertainty is compounded by the dispersion of axons in gray matter and by the u-fiber system in superficial white matter that impedes accurate estimation of tract end points (Reveley et al., 2015).

Despite these challenges, we expect that improvements in the analysis of dMRI will allow ever more powerful inferences to be made about the structure and function of the human visual wiring diagram. For example, by utilizing the modeling of complex fiber organization from dMRI data and improved tractography methods (Reisert et al., 2011; Takemura, Caiafa, Wandell, & Pestilli, 2016) as well as improvements in data resolution and data quality (Sotiropoulos et al., 2013). In these next steps, it will also be important to capitalize on other experimental modalities, such as polarized light imaging (Larsen, Griffin, Gräßel, Witte, & Axer, 2007), that provide high-resolution models of human visual pathways in postmortem measurements.

## Relationship between the visual white matter and visual impairment

Visual deprivation resulting from loss of input from the eye provides an excellent test bed for understanding the links between white matter structure and function and visual function in vivo. This is because of our extensive understanding of the anatomical and functional structure of the visual system and because it allows us to measure the effects of dramatically different experience even as the brain itself remains largely intact. Since the classic studies of Hubel and Wiesel in the 1960s (Wiesel & Hubel, 1963a, 1963b, 1965a, 1965b), visual deprivation has been an important model for examining the effects of early experience on brain development and function. In this section, we focus on the effects of visual loss due to peripheral blindness (such as retinal disease) on white matter pathways. In addition, we discuss how in vivo measurements of white matter structure can provide insight into pathways that mediate blindsight after lesions of the striate cortex. Blindsight provides a particularly elegant example of determining which of multiple candidate pathways are likely to mediate residual behavioral performance based on in vivo measurements of white matter structure.

### White matter changes due to peripheral blindness

A wide variety of studies in animals and humans have demonstrated large-scale functional (Lewis & Fine, 2011), neuroanatomical (Bock & Fine, 2014; Movshon & Van Sluyters, 1981), neurochemical (Coullon, Emir, Fine, Watkins, & Bridge, 2015; Weaver, Richards, Saenz, Petropoulos, & Fine, 2013), and even vascular (De Volder et al., 1997; Uhl, Franzen, Podreka, Steiner, & Deecke, 1993) changes within the occipital cortex as a result of early blindness. Given this extensive literature, peripheral blindness provides a useful model system to examine the reliability and sensitivity of current noninvasive methods for assessing the effects of experience on white matter pathways. Here, we review the white matter changes associated with visual deprivation, paying particular attention to the links between white matter microstructure and alterations of functional responses due to visual loss.

### The optic tract and optic radiations

Early or congenital blindness results in severe degradation of the optic tract (between the eye and the lateral geniculate nucleus) and the optic radiations (between the lateral geniculate nucleus and V1). Not only are these pathways noticeably reduced in volume, but there is also decreased longitudinal and increased radial diffusivity within these tracts (Noppeney, Friston, Ashburner, Frackowiak, & Price, 2005; Reislev, Kupers, Siebner, Ptito, & Dyrby, 2015; Shu et al., 2009), suggesting degradation of white matter microstructure within the remaining tract. These effects seem to be particularly pronounced in anophthalmic individuals (born without eyes), suggesting that retinal signals play a role in the development of these tracts (Bridge, Cowey, Ragge, & Watkins, 2009). Similarly, atrophy of the LGN has consistently been shown in congenital blindness (Cecchetti et al., 2016) and anophthalmia (Bridge et al., 2009). Reductions in FA within the optic tract and optic radiations are also found in individuals who become blind in adolescence or adulthood (Dietrich, Hertrich, Kumar, & Ackermann, 2015; Shimony et al., 2006; Wang et al., 2013), indicating that visual experience is also necessary for maintenance of these tracts. Indeed, there are some indications that the microstructure of these tracts may be more heavily degraded in individuals with acquired blindness than in congenitally blind individuals, but these results may also be confounded with the etiologies of later blindness. For example, glaucoma can cause physical damage to the optic tract.

### Callosal connectivity

Integration of information between the left and right visual fields is mediated by white matter in the splenium, a segment of the posterior corpus callosum. Connections through this part of the corpus callosum can be traced back to various parts of the visual cortex using diffusion-based tractography in sighted individuals (see above; Dougherty et al., 2005).

Studies based on shape analysis of anatomical (T1-weighted) MRI scans and voxel-based morphometry (VBM; Whitwell, 2009), a method that assesses differences in the volume of brain structures in T1-weighted scans using a statistical parametric approach, have reported that early visual deprivation results in a reduction in volume in the posterior portion of the corpus callosum (Leporé et al., 2010; Ptito, Schneider, Paulson, & Kupers, 2008; Tomaiuolo et al., 2014). Meanwhile, studies using dMRI have consistently found reductions in the FA of the splenial portion of the corpus callosum in early blind individuals (Shimony et al., 2006; Yu et al., 2007) and particularly anopthalmic individuals (Bock et al., 2013). However, in contrast to studies based on T1-weighted MRI, Bock et al. (2013) did not find a difference in the volume of the portion of the splenium containing fibers connecting visual areas V1/V2, neither in early blind individuals nor in anophthalmic individuals.

A few explanations can account for these discrepancies. The first are differences in methodology: VBM studies rely on registration to an anatomical atlas to perform their analysis and can confound differences in the volume of a brain structure with changes in tissue composition, consistent with reduced FA found in the dMRI studies. The second explanation stems from the differences in the brain structures studied: The studies based on T1-weighted images anatomically segmented the callosum into subregions of arbitrary sizes without any subject- or modality-specific information. In contrast, Bock et al. (2013) defined the splenium in individual subject data, using a midline sagittal slice and fibers tracked from a surface-based V1/V2 ROI. This strategy results in much more restricted splenial ROIs compared to other studies. It is possible that callosal fibers connecting V1/V2 are less affected by visual deprivation than fibers that represent higher-level cortical areas in other parts of the splenium. This interpretation is supported by fMRI data showing increased lateralization of responses in the occipital cortex beyond early visual areas in congenitally blind individuals (Bedny, Pascual-Leone, Dodell-Feder, Fedorenko, & Saxe, 2011; Watkins et al., 2012). Finally, Shimony et al. (2006) also reported large variability among early blind individuals in terms of the anatomical structures surrounding early visual cortex.

Nevertheless, Bock et al. (2013) also consistently found that the topographic organization of occipital fibers within the splenium is maintained in early blindness even within anophthalmic individuals. It was possible to observe a dorsal/ventral mapping of visual callosal fibers within the splenium, whereby fibers connecting dorsal V1/V2 (representing the lower visual field) project to the superior–posterior portion of the splenium, and fibers connecting ventral V1/V2 (representing the upper visual field) project to the inferior–anterior portion of the splenium. Similarly, an eccentricity gradient was found in the splenium from a foveal representation in the anterior–superior portion of the splenium to a peripheral representation in the posterior–inferior part of the splenium. As expected, this mapping of eccentricity was orthogonal to the dorsal/ventral mapping (Bock et al., 2013).

### Cortico-cortical connections

Over the last two decades, a considerable number of studies have observed functional cross-modal plasticity (novel or augmented responses to auditory or tactile stimuli within the occipital cortex) as a result of congenital blindness. Specifically, occipital regions have been shown to demonstrate functional responses to a variety of auditory (Gougoux et al., 2004; Jiang, Stecker, & Fine, 2014; Lessard, Paré, Lepore, & Lassonde, 1998; Röder et al., 1999; Voss et al., 2004), language (Bedny et al., 2011; Burton, Diamond, & McDermott, 2003; Cohen et al., 1997; Röder, Stock, Bien, Neville, & Rösler, 2002; Sadato et al., 1996; Watkins et al., 2012), and tactile (Alary et al., 2009; Goldreich & Kanics, 2003; Van Boven, Hamilton, Kauffman, Keenan, & Pascual-Leone, 2000) stimuli. One of the more attractive explanations for these cross-modal responses, given the animal literature (Innocenti & Price, 2005; Restrepo, Manger, Spenger, & Innocenti, 2003; Webster, Ungerleider, & Bachevalier, 1991), is that they might be mediated by altered white matter connectivity—for example, a reduction in experience-dependent pruning. As a result, when methods for measuring white matter tracts in vivo became available, there was immediate interest in looking for evidence of novel and/or enhanced connections within early blind individuals. However, despite the massive changes in functional responses within the occipital cortex that are observed as a result of blindness, cortico-cortical white matter changes as a result of early blindness are not particularly dramatic with little or no evidence for enhanced connectivity as predicted by the “reduced pruning” hypothesis.

Indeed, reduced FA is consistently found within white matter connecting the occipital and temporal lobes (Bridge et al., 2009; Reislev et al., 2015; Shu et al., 2009). This finding is somewhat puzzling given that several studies have shown that language stimuli activate both the lateral occipital cortex and fusiform regions in congenitally blind individuals (Bedny et al., 2011; Watkins et al., 2012). Similarly, no increase in FA has been detected in the pathways between the occipital and superior frontal cortex, a network that has shown to be activated by language in congenitally blind populations (Bedny et al., 2011; Watkins et al., 2012), and two studies have found evidence of deterioration (e.g., reduced FA) within the inferior fronto-occipital fasciculus (Reislev et al., 2015; Shu et al., 2009).

It is not clear what causes the lack of consistency in the studies described above. One likely factor is between-subject variability. Estimates of individual differences in tracts suggest that between-group differences require surprisingly large subject numbers even for relatively large and consistently located tracts. For example, within the inferior and superior longitudinal fasciculus, to detect a 5% difference in fractional anisotropy at 0.9 power would require 12–19 subjects/group in a between-subject design (Veenith et al., 2013). Because of the difficulty of recruiting blind subjects with relatively homogenous visual histories, many of the studies cited above used moderate numbers of subjects and may have been underpowered.

One of the more puzzling findings described above is the consistent finding of reduced white matter connectivity between the occipital and temporal lobes and between the occipital and superior frontal lobes given that the occipital cortex shows enhanced responses to language in blind individuals. One possibility is that measured connectivity provides a misleading picture of actual connectivity between these regions. A potential confound is the effect of crossing fibers. If blindness leads to a loss of pruning that extends beyond these major tracts then the resulting increase in the prevalence of crossing fibers within other networks might measurably reduce FA within the occipito-temporal and occipito-frontal tracts.

Alternatively, it is possible that the enhanced language responses found in the occipital cortex genuinely coexist with reduced connectivity with the temporal cortex. For example, we have recently suggested that an alternative perspective in understanding cortical plasticity as a result of early blindness is to consider that the occipital cortex may compete rather than collaborate with nondeprived regions of cortex for functional roles (Bock & Fine, 2014). According to such a model, the lateral occipital cortex may compete with temporal areas with language tasks assigned to the two regions in such a way as to maximize the decoupling between the areas. In the context of such a framework, reductions in anatomical connectivity between the two areas are less surprising.

The effects of early compared to late blindness on cortico-cortical white matter connections remain unclear. One study has found that reductions in FA may be more widespread in late acquired blindness compared to congenital blindness with late blind individuals showing reduced FA in the corpus callosum, anterior thalamic radiations, and frontal and parietal white matter regions (Wang et al., 2013). Such a result is quite surprising although it is possible that the cross-modal plasticity that occurs in early blind individuals might prevent deterioration within these tracts, and this “protective” effect does not occur in late blind individuals, who show far less cross-modal plasticity. However this difference between late and early blind subject groups was not replicated by Reislev et al. (2015), who noted similar losses in FA for early and late blind subjects within both the inferior longitudinal fasciculus and the inferior fronto-occipital fasciculus using a tract-based approach. This group did find significant increases in radial diffusivity within late blind subjects relative to sighted control subjects but did not directly compare radial diffusivity between late and early blind subject groups.

### White matter changes due to cortical blindness

Damage to the primary visual cortex (V1) causes the loss of vision in the contralateral visual field (homonymous hemianopia) and degeneration of the geniculo-striate tract. Despite these lesions and a lack of conscious vision, some individuals can still correctly detect the presence/absence of a stimulus (Poppel, Held, & Frost, 1973) as well as discriminate between variations in stimulus color and motion (Ffytche, Guy, & Zeki, 1996; Morland et al., 1999)—a condition known as “blindsight” (Poppel et al., 1973; Weiskrantz, Warrington, Sanders, & Marshall, 1974). Given the absence of the major geniculo-striate projection, any residual visual information is likely to be conveyed by an intact projection from subcortical visual regions to the undamaged visual cortex.

Although several individual case studies have examined the white matter pathways underlying residual vision (Bridge et al., 2010; Bridge, Thomas, Jbabdi, & Cowey, 2008; Leh, Johansen-Berg, & Ptito, 2006; Tamietto, Pullens, de Gelder, Weiskrantz, & Goebel, 2012), a critical test of the functional relevance of white matter microstructure was recently carried out by comparing individuals with V1 lesions who did and did not demonstrate residual “blindsight” vision. Ajina et al. (2015) examined white matter microstructure within three potential pathways that might subserve residual vision in hemianopia patients. They showed that two potential pathways (superior colliculus to hMT+ and the interhemispheric connections between hMT+) did not differ in microstructure between those with and without blindsight. In contrast, the pathway between LGN and hMT+ in the damaged side differed significantly across individuals with and without blindsight. Patients with blindsight had comparable FA and MD in damaged and intact hemispheres. Patients without blindsight showed a loss of this tract in the damaged hemisphere (Ajina et al., 2015). These results provide an elegant example of how white matter measurements can be correlated with visual function in order to infer structure–function relationships.

### Summary

Within the subcortical pathways, our review of the literature suggests that in vivo measurements of white matter microstructure can provide sensitive and reliable measurements of the effects of experience on white matter. Studies consistently find the expected deterioration within both the optic tract and the optic radiations in the case of peripheral blindness. Similarly, analysis of subcortical pathways in blindsight has shown consistent results across several cases and are interpretable based on the functional and preexisting neuroanatomical literature.

In contrast, it has proved surprisingly difficult to obtain consistent results for measurements of cortico-cortical connectivity across different studies. These discrepancies in the literature are somewhat surprising given the gross differences in visual experience that occurs in early and even late blind individuals. One possible explanation is that, as described above, most studies (although with a few exceptions, e.g., Wang et al., 2013) have tended to have relatively low statistical power. Larger sample sizes (perhaps through a multicenter study) might better reveal more subtle anatomical differences in connectivity as a result of early blindness. A second factor may be methodological differences across studies—early blind subjects are anatomically distinct within occipital cortex—for example, occipital cortex shows less folding in animal models of blindness (Dehay, Giroud, Berland, Killackey, & Kennedy, 1996), which may affect estimates of white matter tracts that are based on methods involving normalization to a canonical template. Comparing results based on tracts identified based on normalization to anatomical templates to those obtained using tracts identified within individual anatomies may provide some insight into how these very different approaches compare in terms of sensitivity and reliability.

It has also proved surprisingly difficult to interpret alterations in white matter cortico-cortical connectivity in the context of the functional literature; occipito-temporal and occipito-frontal white matter connections have consistently been shown to be weaker in early blind subjects despite the apparent recruitment of occipital cortex for language. This discrepancy between estimates of white matter connectivity and functional role within cortico-cortical tracts makes it clear that drawing direct conclusions from white matter microstructure to functional role is still fraught with difficulty. This is not a reason to abandon the enterprise but rather provides a critical challenge. To return to the argument with which we began this chapter, the effects of visual deprivation provide an excellent model system for testing how well we understand the measurement and interpretation of in vivo measurements of white matter infrastructure.

## Properties of white matter circuits involved in face perception

**Figure 7:**
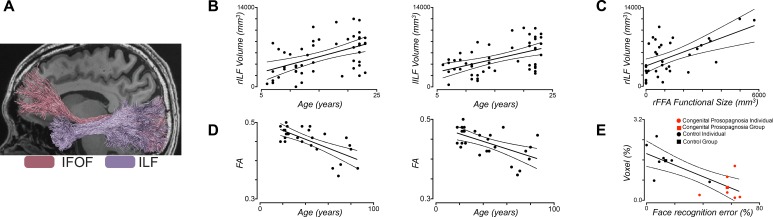
The role of the inferior longitudinal fasciculus (ILF) and the inferior fronto-occipital fasciculus (IFOF) in face perception. (A) Representative example of fibers extracted from the ILF, which is the primary occipitotemporal associative tract (Crosby, 1962; Gloor, 1997) that propagates signals through the ventral visual cortex between the primary visual cortex and the anterior temporal lobe and, finally, from the IFOF, which begins in the occipital cortex, continues medially through the temporal cortex dorsal to the uncinate fasciculus, and terminates in the inferior frontal and dorsolateral frontal cortex (Catani et al., 2002). (B) In typically developing individuals, there is a systematic age-related increase in the macrostructural properties of both the right and left ILF, which is measured here by the volume of the tract in cubic millimeters across the first two decades of life. (C) We observed a joint structure–function correspondence between the size of the individually defined functional right FFA and the volume of the right ILF, which held even when age was accounted for (Scherf et al., 2014). (D) In typically developing adults, there is a systematic age-related decline in the microstructural properties of both the right and left IFOF as measured by the mean FA, indicating that it is disproportionately vulnerable compared to the ILF during the aging process. (E) Individuals who have a lifetime history of face blindness, an inability to recognize faces, in spite of normal intelligence and sensory vision, have systematically smaller volume visual fiber tracts, particularly in the right ILF, as depicted here, compared to age-matched control participants. Together, these findings provide converging evidence that these two major tracts, the IFOF and the ILF, carry signals important for face perception.

In the visual system, neural signals are transmitted through some of the most prominent long-range fiber tracts in the brain: The optic radiations splay out from the thalamus to carry visual signals to the primary visual cortex; the forceps major, u-shaped fibers that traverse the splenium of the corpus callosum innervate the occipital lobes, allowing them to integrate neural signals; the inferior longitudinal fasciculus (ILF) is the primary occipito-temporal associative tract (Crosby, 1962; Gloor, 1997) that propagates signals through the ventral visual cortex between the primary visual cortex and the anterior temporal lobe (see Figure 7A); and, finally, the inferior fronto-occipital fasciculus (IFOF) begins in the occipital cortex, continues medially through the temporal cortex dorsal to the uncinate fasciculus, and terminates in the inferior frontal and dorsolateral frontal cortex (see Figure 7A; Catani et al., 2002). Increasingly, diffusion neuroimaging studies are providing evidence indicating the importance of these white matter tracts for visual behavior. Here, we present evidence from converging streams of our research demonstrating that the structural properties of the ILF and IFOF are critically important for intact face perception.

Face perception is a complex suite of visual behaviors that is subserved by a distributed network of neural regions, many of which are structurally connected by the ILF and IFOF. For example, the “core” or posterior regions include the occipital face area (OFA), the fusiform face area (FFA), and the posterior superior temporal sulcus, and the “extended” areas include the anterior temporal pole, amygdala, and ventro-medial prefrontal cortex (Gobbini & Haxby, 2007). Recently, functional neuroimaging studies have provided supporting, albeit indirect, evidence of rapid interactions between these posterior core regions and the extended anterior regions (e.g., anterior temporal lobe and amygdala) that implicate the involvement of long-range association fiber tracts that connect these regions, including the ILF and the IFOF (Bar et al., 2006; Gschwind, Pourtois, Schwartz, Van De Ville, & Vuilleumier, 2012; Rudrauf et al., 2008; Song et al., 2015).

Finally, damage to either of these pathways disrupts face processing (Catani, Jones, Donato, & Ffytche, 2003; Catani & Thiebaut de Schotten, 2008; Fox, Iaria, & Barton, 2008; Philippi, Mehta, Grabowski, Adolphs, & Rudrauf, 2009; Thomas et al., 2009), suggesting that these white matter tracts serve as a critical component of the neural system necessary for face processing. In what follows, we provide evidence showing that age-related changes in the structural properties of these tracts in early development is associated with emergent properties of the functional neural network supporting face processing. We also review evidence that age-related decreases in the structural properties of these tracts in aging adults exist and are associated with decrements in face processing behavior. Finally, we review data showing that relative deficits in the structural properties of these long-range fiber tracts potentially explain the causal nature of face blindness in individuals with congenital prosopagnosia. Together these findings converge to indicate that the structural properties of white matter tracts, and the ILF and IFOF in particular, are necessary for skilled face perception.

Across all of these studies, we use a common methodological approach. We acquire diffusion images, analyze them using a tensor model, and perform deterministic tractography using the fiber assignment of continuous tracking algorithm and brute-force fiber reconstruction approach (Mori et al., 1999; Xue, van Zijl, Crain, Solaiyappan, & Mori, 1999) with fairly standard parameters. To extract the tracts of interest, we use a multiple ROI approach that is very similar to Wakana et al. (2007). From each tract, we extract the volume (the number of voxels through which the fibers pass multiplied by the volume of the voxel) as well as the mean FA, MD, axial diffusivity, and radial diffusivity values across these voxels.

### Age-related changes in the ILF and IFOF with functional neural change

The central question guiding this work was whether developmental differences in the structural properties of the ILF and IFOF, defined independently using anatomical ROIs, are related to developmental differences in the characteristics of the functional face-processing regions connected by these tracts (Scherf, Thomas, Doyle, & Behrmann, 2014). Across participants whose ages covered a substantial range (ages 6–23 years), we evaluated differences in (a) the macro- and microstructural properties of the fasciculi and (b) the functional profile (size, location, and magnitude of selectivity) of the face- and place-selective regions that are distributed along the trajectory of the pathways of interest using fMRI. First, we found that all tracts, with the exception of the left IFOF, exhibited age-related improvements in their microstructural properties, evincing a significant decrease in mean and radial but not axial diffusivity from childhood to early adulthood (see Figure 7B). This result is consistent with the idea that the increasingly restricted diffusion perpendicular to the axons in these tracts reflects continued myelination from childhood through early adulthood. The left IFOF exhibited stable levels of microstructural properties across the age range, indicating that it may be a very early developing fiber tract. At the macrostructural level, only the ILF exhibited an age-related change in volume, which was evident in both hemispheres. The combined increases in microstructural properties and the volume of the right ILF suggest that it is becoming increasingly myelinated and/or more densely packed with axons with age (Beaulieu, 2002; Lebel & Beaulieu, 2011; Song et al., 2005).

Having identified the structural changes that potentially contribute to circuit organization, we explored concomitant differences in the functional profile of face-related cortical regions and then examined the joint structure–function correspondences. Across the full age range, individuals with larger right FFA volumes also exhibited larger right ILF volumes (see Figure 7C). Neither the right OFA nor the right PPA exhibited this structure–function relationship with the right ILF. This structure–function relationship between the right FFA and right ILF was also present in just the children and adolescents (aged 6–15 years). However, once age was accounted for in the same model as the size of the right FFA in the children and adolescents, the age effects on the volume of the ILF swamped all the significant variation even though the size of the right FFA increased significantly across this age range. One interpretation of these findings is that the neural activity generated by larger functional regions may require and/or influence the development of larger fiber tracts (via increasing myelination of existing axons and/or more densely packed axons) to support the transmission of neural signals emanating from such regions. It may also be that increasing the integrity of the structural architecture of fiber tracts increases the propagation of the neural signal throughout the circuit, thereby enhancing the functional characteristics of the nodes within the circuit (and vice versa). In other words, structural refinements of white matter tracts may precede and even be necessary for functional specialization of the circuit to emerge. In sum, this work uncovers the key contributions of the ILF and IFOF and their relationship to functional selectivity in the developing circuitry that mediates face perception.

### Aging-related decrease in structural properties of IFOF with face perception deficits

Although the findings above focus on understanding how the structural properties of the ILF and IFOF are critical for building the complex face-processing network during early development, it was also important to understand whether disruptions in these white matter tracts might be associated with or even responsible for age-related declines in face processing that are well reported (e.g., Salthouse, 2004) and are not solely a function of memory or learning changes (Boutet & Faubert, 2006). We scanned 28 individuals aged 18–86 years using a diffusion tensor imaging protocol (Thomas et al., 2008). We also tested them in face and car perceptual discrimination tasks. We observed that the right IFOF was the only tract that decreased in volume (as measured by percentage of fibers and voxels through which the fibers pass) as a function of age. In contrast, the bilateral IFOF and the left but not right ILF exhibited age-related decreases in FA (see Figure 7D). To summarize, it is the IFOF in the right hemisphere that shows particular age-related vulnerability although there is a tendency for the tracts in the left hemisphere to show some reduction in microstructural properties as revealed in FA values as well.

On the discrimination tasks, participants performed more poorly in the difficult trials, especially in the face compared to the car condition. Of relevance though is that the older individuals, the 60- and 80-year-olds, made significantly more errors on faces than they did on cars, and performance was almost at chance in the difficult condition. Given that there were age-related declines in the micro- and macrostructural properties of the IFOF as well as in face perception behavior, we also explored whether there was an association between these tract and behavioral deficits. To address this question, we examined correlations between behavioral performance and the normalized percentage of fibers, normalized percentage of voxels, and average FA values in the ILF and IFOF in each hemisphere. We observed two main findings: (a) During the easy face trials, participants with a greater percentage of fibers in the right IFOF exhibited better performance in these easy discriminations, and (b) during the difficult face trials, participants with higher FA values and larger volume right IFOF exhibited better performance on the difficult discriminations. Taken together, these findings indicate a clear association between the ability to discriminate between faces and the macro- and microstructural properties of the IFOF in the right hemisphere.

### Disruptions in ILF and IFOF may explain “face blindness”

Finally, given the findings of an association between face processing behavior and the structural properties of the IFOF in typically developing adults, we investigated whether congenital prosopagnosia, a condition that is characterized by an impairment in the ability to recognize faces despite normal sensory vision and intelligence, might arise from a disruption to either and/or both the ILF and IFOF (Thomas et al., 2009). This hypothesis emerged following empirical findings that the core functional neural regions, including the FFA, appeared to produce normal neural signals in many congenital prosopagnosic individuals (Avidan, Hasson, Malach, & Behrmann, 2005; Avidan et al., 2014; Thomas et al., 2009). To test this hypothesis, we scanned six adults with congenital prosopagnosia, all of whom evinced normal BOLD activation in the core face regions (Avidan et al., 2005), and 17 age- and gender-matched control adults. We also measured participants' face recognition skills. Relative to the controls, the congenital prosopagnosia group showed a marked reduction in both the macro- and microstructural properties of the ILF and IFOF bilaterally. We then carried out a stepwise regression analysis with the connectivity and behavioral measures. Across participants, individuals with the poorest face recognition behavior had the lowest FA and the smallest volume in the right ILF (see Figure 7E). Similarly, poor face recognition behavior was also related to smaller volume of the right IFOF as well. In summary, our study revealed that the characteristic behavioral profile of congenital prosopagnosia may be ascribed to a disruption in structural connectivity in some portion of the ILF and, perhaps to a lesser extent, the IFOF as well (for related findings, see Gomez et al., 2015; Song et al., 2015).

### Summary

We have presented converging evidence that face recognition is contingent upon efficient communication across disparate nodes of a widely distributed network, which are connected by long-range fiber tracts. Specifically, we have shown that two major tracts, the IFOF and the ILF, carry signals important for face perception. Studies in children and older individuals as well as investigations with individuals who are impaired at face recognition all attest to the required integrity of the tracts for normal face recognition behavior. Early in development, the ILF undergoes a particularly long trajectory in which both the micro- and macrostructural properties change in ways indicative of increasing myelination. Of relevance for the integrity of face perception behavior, there is a highly selective and tight correspondence between age-related growth in one of the preeminent functional nodes of the face-processing neural network, the right FFA, and these age-related improvements in the structural properties of the ILF. These findings reflect the dynamic and intimate nature of the relationship between brain structure and function, particularly with respect to the role of white matter tracts, in setting up neural networks that support complex behaviors such as face perception. Our work with congenital prosopagnosic participants suggests that face-processing behavior will not develop normally when this developmental process is disrupted. Future work will need to identify when, developmentally, white matter disruptions are present and interfere with face perception. Finally, our research in aging adults indicates that the IFOF is particularly vulnerable over the course of aging, which can lead to difficulties with face recognition behavior. However, Grossi et al. (2014) reported that an adult with progressive prosopagnosia, a gradual and selective inability to recognize and identify faces of familiar people, had markedly reduced volume in the right but not left ILF whereas the bilateral IFOF tracts were preserved. This suggests that there may be some conditions in adulthood in which the ILF is also vulnerable. Together, these findings converge on the claim that the structural properties of white matter circuits are, indeed, necessary for face perception.

## Conclusions and future directions

The goal of this review was to survey a range of findings that demonstrate the specific importance of studying the white matter of the visual system. We focused on findings in human brains that used the only currently available method to study the white matter in vivo in humans: dMRI. In the time since its inception in the 1990s, dMRI methods have evolved, and evidence about the importance of the white matter has accumulated (Fields, 2008b). These findings are modern, but they support a point of view about the nervous system that has existed for a long time. Connectionism, the theory that brain function arises from its connectivity structure, goes back to classical work of the 19th-century neurologists (Deacon, 1989). Over the years, interest in disconnection syndromes waxed and waned as more holistic views of the brain (Lashley, 1963) and more localizationist views of the brain prevailed. As a consequence, systems neuroscience has traditionally focused on understanding the response properties of individual neurons and cortical regions (Fields, 2004, 2013). Only a few studies were devoted to understanding the relationship of the white matter to cognitive function. Similar to electrical cables, it was believed that the connections in the brain were either intact and functioning or disconnected. However, the pendulum started swinging back dramatically toward connectionism already in the 1960s (Geschwind, 1965), and over the years, there has been increasing appreciation for the importance of brain networks in cognitive function, culminating in our current era of connectomics (Sporns et al., 2005). In addition to an increasing understanding of the importance of connectivity, views that emphasize the role of tissue properties not related to neuronal firing in neural computation have more recently also evolved and come to the fore (Bullock et al., 2005; Fields, 2008b)—for example, the role of glial cells in modulating synaptic transmission and neurotransmitter metabolism as well as the role of the white matter in metabolism and neural hemodynamic coupling (Robel & Sontheimer, 2016). Today, we know much more about the white matter than ever before, and we understand that the properties of the tissue affect how communication between distal brain areas is implemented. dMRI enables inferences about the properties of the white matter tissue in vivo, which in turn enables inferences about the connection between behavior and biology.

As we have seen in the examples presented above, dMRI is a useful method to study the biological basis of normal visual perception and to glean understanding about the connectivity that underlies the organization of the visual cortex, about the breakdowns in connectivity that occur in different brain disorders, and about the plasticity that arises in the system in response to visual deprivation. But although the findings reviewed above demonstrate the importance of the white matter in our understanding of the visual system and the biological basis of visual perception, they also serve as a demonstration of the unique capacity of vision science to study a diverse set of phenomena across multiple levels of description. This is largely an outcome of the detailed understanding of different parts of the visual system and attributable to the powerful quantitative methods that have developed in the vision sciences to study the relationship between biology and perception. For these reasons, the visual system has historically proven to be a good testing ground for new methodologies. Another recurring theme of the examples presented above is the importance of convergent evidence in cognitive neuroscience (Ochsner & Kosslyn, 1999). Studies of the human visual white matter exemplify this: Evidence from behavior, from fMRI, from developmental psychology, and from physiology converge to grant us a unique view about the importance of specific pathways for perception. The response properties of different brain regions are heavily influenced by the synaptic inputs of long-range connections. For example, one might grow to better understand the response properties of dorsal visual areas when their connection through the white matter with ventral visual areas is known (see the section “Tracts and connections across human visual maps”). Similarly, the role of different functional regions in the face perception network becomes clearer when the relationship between the size of these regions, the size of the ILF, and the codevelopment of these anatomical divisions is demonstrated (see the section “Properties of white matter circuits involved in face perception”). The study of individuals with perceptual deficits or with visual deprivation demonstrates the specific importance of particular connections and delineates the lifelong trajectory of the role of these trajectories, also revealing possibilities and limitations of brain plasticity (see the section “Relationship between the visual white matter and visual impairment”). The importance of converging evidence underscores the manner in which understanding the white matter may continue to have an effect on many other parts of cognitive neuroscience even outside of the vision sciences.

Two major difficulties recur in the descriptions of findings reviewed above: The first is an ambiguity in the interpretation of findings about connectivity. The section “Methods for in vivo study of the human white matter” described some of the limitations we currently have in validating tractography solutions. The resolution of the measurement together with challenges in the analysis of the data limit our ability to discover new tracts with tractography and may limit the interpretation of individual differences in connectivity. Recent reports demonstrated that increasing the resolution of the data may be only part of the story. Unless high-quality data are also associated with an optimal choice of tracking methods, important and known connections can be missed (Takemura, Pestilli et al., 2016; Thomas et al., 2014). The dilemma is that the best tractography analysis method likely depends on the properties of the data as well as the fascicle to be estimated. For the time being, no fixed set of rules will work in every case (Takemura, Caiafa et al., 2016). Nevertheless, finding major well-known tracts in individual humans is not a major problem with current technology and can even be fully automated (Kammen et al., 2015; Yeatman et al., 2012; Yendiki et al., 2011).

The other difficulty relates to the interpretation of microstructural properties, such as FA and MD. Although these relate to biophysical properties of the tissue in the white matter, they contain inherent ambiguities. For example, FA may be higher in a specific location in one individual relative to another because of an increased density of myelin in that individual, but it may also be higher because of a decrease in the abundance of tracts crossing through this region. In the following last section, we outline a few directions that we believe will influence the future of the field and perhaps help address some of these difficulties.

### Future directions

One of the common threads across the wide array of research in human vision science using dMRI and a source of confusion in evaluating the dMRI literature is the ambiguity in interpretation: Changes in measured dMRI parameters may be caused by different biological processes. Some of the promising directions that we hope will reduce these ambiguities in future research have to do with new developments in the measurements and modeling of MRI data in human white matter. These developments will also build upon increased openness in dMRI research: the availability of large, publicly available datasets and open source software to implement a wealth of approaches to the analysis of these data.

#### Biophysical models and multi b-value models

When more than one diffusion weighting b-value is collected, additional information about tissue microstructure can be derived from the data. This includes compartment models that account for the signal as a combination of tensors (Clark & Le Bihan, 2000; Mulkern et al., 1999) or extends the Gaussian tensor model with additional terms (so-called diffusion kurtosis imaging; Jensen, Helpern, Ramani, Lu, & Kaczynski, 2005). Other models consider the diffusion properties of different kinds of tissue. For example, the CHARMED model (Assaf & Basser, 2005) explicitly computes the contributions of intra- and extracellular water to the signal. Other models describe axon diameter distribution (Alexander et al., 2010; Assaf, Blumenfeld-Katzir, Yovel, & Basser, 2008) and dispersion and density of axons and neurites (Zhang, Schneider, Wheeler-Kingshott, & Alexander, 2012). The field is currently also struggling with the ambiguity of these models (Jelescu, Veraart, Fieremans, & Novikov, 2016), and this is a very active field of research (Ferizi et al., 2016). The ultimate goal of these efforts is to develop methods and models that help infer physical quantities of the tissue that are independent of the measurement device. This goal can already be achieved with other forms of MRI measurements, which we discuss next.

#### Combining diffusion-weighted and quantitative MRI

Quantitative MRI refers to an ever-growing collection of measurement methods that quantify the physical properties of neural tissue, such as its density (Mezer et al., 2013) or molecular composition (Stüber et al., 2014). These methods have already been leveraged to understand the structure and properties of the visual field maps (Bridge, Clare, & Krug, 2014; Sereno, Lutti, Weiskopf, & Dick, 2013). Combinations of these methods with dMRI can help reduce the ambiguity and provide even more specific information about tissue properties in the white matter (Assaf et al., 2013; Mezer et al., 2013; Mohammadi et al., 2015; Stikov et al., 2015; Stikov et al., 2011). For example, a recent study used this combination to study the biological basis of amblyopia (Duan et al., 2015).

#### Large open datasets

The accumulation of large open datasets with thousands of participants will enable the creation of models of individual variability at ever-finer resolution (Pestilli, 2015). This will provide more confident inferences about the role of white matter in vision. The Human Connectome Project (Van Essen et al., 2013) is already well on its way to providing a dataset encompassing more than a thousand participants, including not only high-quality dMRI data, but also measurements of fMRI of the visual system. Another large, publicly available dataset that includes measurements of both dMRI and fMRI of visual areas is the Enhanced Nathan Klein Institute Rockland Sample (Nooner et al., 2012). Crucially, the aggregation of large datasets can be scaled many times if a culture of data sharing pervades a larger portion of the research community (Gorgolewski & Poldrack, 2016) and as the technical and social tools that allow data sharing become more widespread (Gorgolewksi et al., 2016; Wandell, Rokem, Perry, Schaefer, & Dougherty, 2015).

#### Open source software for reproducible neuroscience

One of the challenges facing researchers that are using dMRI is the diversity of methods available to analyze the data. To facilitate the adoption of methods and the comparison between different methods, transparency of these methods is crucial. Transparency is also crucial in progress toward reproducible science (Donoho, 2010; McNutt, 2014; Stodden, Leisch, & Peng, 2014; Yaffe, 2015).

To facilitate transparency, robust and well-documented open source implementations of the methods have become important. There are several open source projects focused on dMRI, and we review only a selection here: the FMRIB Software Library implements useful preprocessing tools (Andersson & Sotiropoulos, 2016) as well as individual voxel modeling (Behrens et al., 2003) and probabilistic tractography (Behrens et al., 2007). The MRtrix library implements novel methods for analysis of individual data and group analysis (Tournier et al., 2012). Vistasoft, mrTools, AFQ, and LiFE (Dougherty et al., 2005; Hara, Pestilli, & Gardner, 2014; Pestilli, Carrasco, Heeger, & Gardner, 2011; Pestilli et al., 2014; Yeatman et al., 2012) integrate tools for analysis of visual fMRI with tools for dMRI analysis and tractography segmentation. Camino provides a set of tools with a particular focus on multi b-value analysis. Dipy (Garyfallidis et al., 2014) capitalizes on the vibrant scientific Python community (Perez, Granger, & Hunter, 2010) and the work of the Neuroimaging in Python community (Nipy; Millman & Brett, 2007) to provide implementations of a broad array of dMRI methods, ranging from established methods to newer methods for estimation of microstructural properties (Fick, Wassermann, Caruyer, & Deriche, 2016; Jensen & Helpern, 2010; Özarslan, Koay, & Basser, 2013; Portegies et al., 2015) and methods for tract clustering and tract registration (Garyfallidis et al., 2012; Garyfallidis et al., 2015) as well methods for statistical validation of dMRI analysis (Pestilli et al., 2014; Rokem et al., 2015).

As the number and volume of available datasets grow large, another promising direction is the adoption of approaches from large-scale data analysis in neuroscience (Caiafa & Pestilli, 2015; Freeman, 2015; Mehta et al., 2016). These methods will enable more elaborate and computationally demanding models and methods to be considered in the analysis of large, multiparticipant dMRI datasets.

## Supplementary Material


