# Supplementary figures and images for: The visual white matter: The application of diffusion MRI and fiber tractography to vision science
Source: J Vis. 2017 Feb 14;17(2):4. doi: 10.1167/17.2.4 (PMC5317208; doi:10.1167/17.2.4)

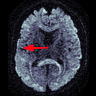

Supplement: Supplementary file 1 [file JOV-05449-2016-s01-ICON.gif]
